# Familial Young-Onset Diabetes, Pre-Diabetes and Cardiovascular Disease Are Associated with Genetic Variants of *DACH1* in Chinese

**DOI:** 10.1371/journal.pone.0084770

**Published:** 2014-01-20

**Authors:** Ronald Ching Wan Ma, Heung Man Lee, Vincent Kwok Lim Lam, Claudia Ha Ting Tam, Janice Siu Ka Ho, Hai-Lu Zhao, Jing Guan, Alice Pik Shan Kong, Eric Lau, Guozhi Zhang, Andrea Luk, Ying Wang, Stephen Kwok Wing Tsui, Ting Fung Chan, Cheng Hu, Wei Ping Jia, Kyong Soo Park, Hong Kyu Lee, Hiroto Furuta, Kishio Nanjo, E. Shyong Tai, Daniel Peng-Keat Ng, Nelson Leung Sang Tang, Jean Woo, Ping Chung Leung, Hong Xue, Jeffrey Wong, Po Sing Leung, Terrence C. K. Lau, Peter Chun Yip Tong, Gang Xu, Maggie Chor Yin Ng, Wing Yee So, Juliana Chung Ngor Chan

**Affiliations:** 1 Department of Medicine and Therapeutics, The Chinese University of Hong Kong, The Prince of Wales Hospital, Shatin, Hong Kong SAR, People’s Republic of China; 2 Hong Kong Institute of Diabetes and Obesity, The Chinese University of Hong Kong, Hong Kong SAR, People’s Republic of China; 3 Li Ka Shing Institute of Health Science, The Chinese University of Hong Kong, The Prince of Wales Hospital, Shatin, Hong Kong SAR, People’s Republic of China; 4 School of Biomedical Science, The Chinese University of Hong Kong, The Prince of Wales Hospital, Shatin, Hong Kong SAR, People’s Republic of China; 5 School of Life Science, The Chinese University of Hong Kong, The Prince of Wales Hospital, Shatin, Hong Kong SAR, People’s Republic of China; 6 Department of Endocrinology and Metabolism, Shanghai Diabetes Institute, Shanghai Key Laboratory of Diabetes Mellitus, Shanghai Clinical Center for Diabetes, Shanghai Key Clinical Center for Metabolic Disease, Shanghai Jiao Tong University Affiliated Sixth People’s Hospital, Shanghai, People’s Republic of China; 7 Department of Molecular Medicine and Biopharmaceutical Sciences, Graduate School of Convergence Science and Technology and Department of Internal Medicine, College of Medicine, Seoul National University, Chongno-gu, Seoul, Korea; 8 First Department of Medicine, Wakayama Medical University, Wakayama, Japan; 9 Department of Epidemiology and Public Health, National University of Singapore, Singapore, Singapore; 10 Department of Chemical Pathology, The Chinese University of Hong Kong, The Prince of Wales Hospital, Shatin, Hong Kong SAR, People’s Republic of China; 11 Department of Orthopaedics and Traumatology, The Chinese University of Hong Kong, The Prince of Wales Hospital, Shatin, Hong Kong SAR, People’s Republic of China; 12 Department of Biochemistry, Hong Kong University of Science and Technology, Hong Kong SAR, People’s Republic of China; Central China Normal University, China

## Abstract

In Asia, young-onset type 2 diabetes (YOD) is characterized by obesity and increased risk for cardiovascular disease (CVD). In a genome-wide association study (GWAS) of 99 Chinese obese subjects with familial YOD diagnosed before 40-year-old and 101 controls, the T allele of rs1408888 in intron 1 of *DACH1*(Dachshund homolog 1) was associated with an odds ratio (OR) of 2.49(95% confidence intervals:1.57–3.96, *P* = 8.4×10^−5^). Amongst these subjects, we found reduced expression of *DACH1* in peripheral blood mononuclear cells (PBMC) from 63 cases compared to 65 controls (*P* = 0.02). In a random cohort of 1468 cases and 1485 controls, amongst top 19 SNPs from GWAS, rs1408888 was associated with type 2 diabetes with a global *P* value of 0.0176 and confirmation in a multiethnic Asian case-control cohort (7370/7802) with an OR of 1.07(1.02–1.12, *P_meta_* = 0.012). In 599 Chinese non-diabetic subjects, rs1408888 was linearly associated with systolic blood pressure and insulin resistance. In a case-control cohort (n = 953/953), rs1408888 was associated with an OR of 1.54(1.07–2.22, *P* = 0.019) for CVD in type 2 diabetes. In an autopsy series of 173 non-diabetic cases, TT genotype of rs1408888 was associated with an OR of 3.31(1.19–9.19, *P* = 0.0214) and 3.27(1.25–11.07, *P* = 0.0184) for coronary heart disease (CHD) and coronary arteriosclerosis. Bioinformatics analysis revealed that rs1408888 lies within regulatory elements of *DACH1* implicated in islet development and insulin secretion. The T allele of rs1408888 of *DACH1* was associated with YOD, prediabetes and CVD in Chinese.

## Introduction

Genome-wide association studies (GWAS) and their meta-analyses have discovered novel loci for T2D in European [1] and Asian populations with odds ratios (ORs) of 1.1–1.4 [2], [3], [4]. In Asia, the most rapid increase in diabetes occurs in the young-to-middle aged group. Using Hong Kong Chinese as an example, 20% of people with diabetes were diagnosed before the age of 40 years. In these young patients, less than 10% had type 1 presentation and 15% had monogenic diabetes. In the remaining patients, family history, obesity and premature cardiovascular disease (CVD) were prominent features [5], [6], [7]. In the Hong Kong Family Diabetes Study (HKFDS) which recruited family members of patients with young-onset diabetes (YOD), we reported strong heritability of diabetes and obesity [8] with co-linkage of related traits to multiple chromosomal regions including chromosome 1q [9]. However, the genetic basis of this form of YOD has not been studied.

Epidemiological analysis has confirmed the clustering of metabolic syndrome, insulin resistance, diabetes and CVD [10], [11], which may share common genetic, environmental or lifestyle factors [1]. In a meta-analysis of 3 GWAS conducted in Chinese from Hong Kong and Shanghai, we discovered rs10229583 located in 7q32 near *PAX4* which was subsequently confirmed in Asian and Caucasian populations [12]. In the first discovery cohort of this meta-analysis consisting of 99 Hong Kong Chinese patients with YOD diagnosed before 40-year-old with at least 1 affected first degree relative and obesity, the T allele of rs1408888 in intron 1 of *DACH1* (Dachshund homolog 1) with *P*<10^−5^ was replicated in a multiethnic Asian case-control cohort, albeit insignificant in Caucasians.

In *Drosophila, Dachshund* (*DAC)* is the homolog of *DACH1* in human which is a highly conserved transcription factor implicated in developmental biology [13], [14]. In a recent report, *DAC* was found to interact physically with *PAX6* to control insulin expression [14]. In light of these findings, we revisited the risk association of rs1408888 of *DACH1* and explored whether this genetic variant might be associated with YOD and related traits in Chinese populations. To test this hypothesis, we examined the differential expression of *DACH1* in peripheral blood mononuclear cells (PBMC) in patients with YOD and tested the genetic associations of rs1408888 in multiple case-control cohorts followed by bioinformatic analysis. We found reduced expression of *DACH1* in PBMC from subjects with YOD and association of the T allele of rs1408888 with YOD, prediabetes and CVD. This risk variant is located within the vicinity of conserved non-coding elements (CNE) of *DACH1* associated with multiple consensus transcription factor binding sites and chromatin modification sites suggesting possible regulatory functions. These findings suggested that genetic variants of *DACH1* may be implicated in abnormal islet biology resulting in prediabetes, YOD and CVD in Chinese populations.

## Results

### Associations with familial young-onset T2D in GWAS

**Figure 1:**
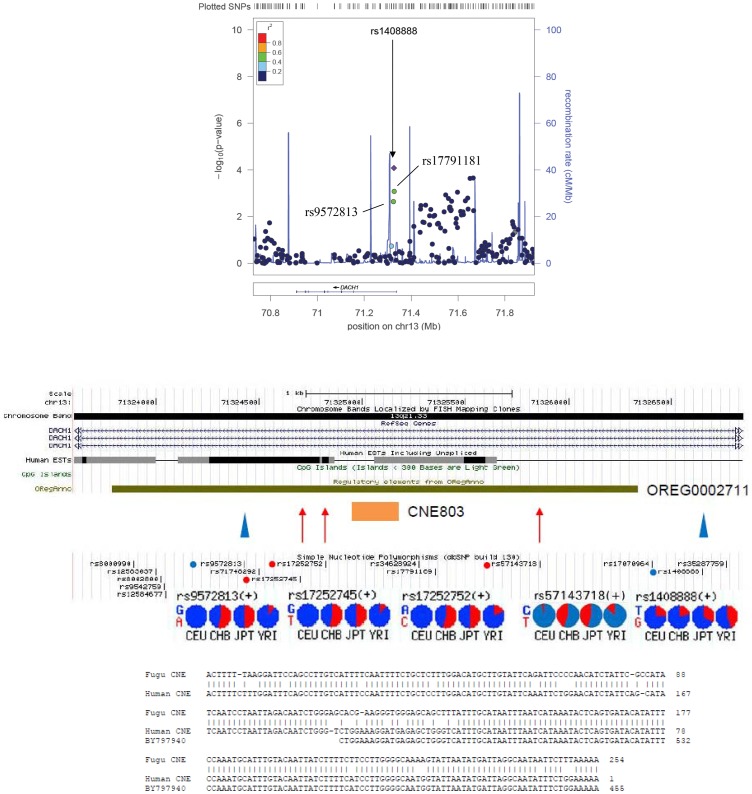
Upper panel: Regional plot showing significant association of rs1408888 in the *DACH1* locus. The –log_10_ *P* values for the allelic test from stage 1 (genome scan) were plotted as a function of genomic position (NCBI build 36). Rs1408888 which showed the strongest signal and neighboring genotyped SNPs in the joint analysis were denoted by purple diamond. LD information (based on HapMap) was shown by color-coded points. Two neighboring SNPs rs9572813 and rs17791181, which showed nominal significance and moderate linkage disequilibrium (0.4 < r^2^ < 0.6) with rs1408888 were indicated. Estimated recombination rate (the blue line) based on the Japanese and Chinese HapMap population was plotted to reflect the local LD structure around the significant SNPs. Gene annotations were taken from NCBI. **Lower panel:** Bioinformatics analysis of genomic region surrounding rs1408888. The region harboring rs1408888 lies in close vicinity of 2 highly conserved non-coding elements, CNE803 and OREG0002711. The two blue arrowheads at the end indicate the positions of rs1408888 (right blue dot) and rs9572813 (left blue dot). The three internal red arrows indicate the positions of the three SNPs (rs17252745, rs17252752 and rs57143718, red dots from left to right) genotyped by sequencing. The allele frequencies of the SNPs are shown in pie chart at the bottom. The alignment of the highly conserved fugu CNE803, the human sequence corresponding to the fugu CNE and the eye prepared EST BY797940 are also shown.

In the GWAS of 99 obese Chinese subjects with familial YOD and 101 controls, 425,513 of 541,891 autosomal SNPs passed quality control with no population stratification using multidimensional scaling analysis and after adjusting for genomic control (GC). From stage 1 GWAS, 24 unique loci with the lowest *P* value in the dataset (*P*<10^−4^) were taken forward for replication in 1468 cases and 1485 controls. Of these, 19 SNPs passed the quality control criteria and 2 SNPs (rs1408888 and rs1449675) remained significantly associated with T2D (Table 1). The intronic SNP rs1408888 {stage 1: OR [95% confidence intervals (CI)] = 2.49(1.57–3.96), *P* = 8.4×10^−5^; stage 2: 1.15(1.03–1.29), *P* = 0.0164} was located at chromosome 13q21.3 and lies within the first intron of the *DACH1* gene (Figure 1). The intergenic SNP rs1449675 [stage 1: OR = 5.33(2.30–12.36); *P* = 2.0×10^−5^, 1.19(1.00–1.41); *P* = 0.0439] was located at chromosome 6q25.3. In the combined analysis (1567 cases, 1586 controls), three more SNPs (rs6595551 in *ZNF608*, rs987105 in *MUT*, and rs1413119 in an intergenic region on chromosome 13) showed nominal associations (*P*<0.05). Among these five SNPs, rs1408888 in *DACH1* had the highest OR [1.21(1.08–1.35); *P* = 9.1×10^−4^] which remained significant (*P* = 0.0176) after correction for multiple testings of the 19 SNPs using 10,000 permutations. In a meta-analysis of 5 Asian case-control cohorts consisting of 7370 cases and 7802 controls, we obtained a combined OR of 1.07(1.02–1.12, *P* = 0.0112) with no heterogeneity [*P* = 0.107 in Cochran’s *Q* test and *I* ^2^ = 44.8% (0.0%–78.1%)] (Table 2).

**Table 1:** Association of SNPs with familial young-onset Type 2 diabetes and obesity in Hong Kong Chinese in a genome-wide association study using Illumina HumanHap550 chip with *P* values less than 10^−4^.

										Joint analysis of stage 1 + 2		
SNP	Chr.	*Nearest gene(s)	Risk allele	Stage	RAF (T2D)	RAF (Controls)	HapMap CHB RAF	OR (95% CI)	*P*_Allelic_	OR (95% CI)	Combined *P*_Allelic_	*P*_permutation_
rs841859	1	SLC2A1	G	1	0.237	0.089	0.173	3.18 (1.77 – 5.71)	5.8 × 10^−5^	1.12 (0.97 – 1.29)	0.1386	0.9422
				2	0.134	0.131		1.03 (0.89 – 1.2)	0.6868			
rs6661853	1	CNIH3	G	1	0.798	0.614	0.673	2.48 (1.59 – 3.89)	5.4 × 10^−5^	0.97 (0.87 – 1.09)	0.6128	1.0000
				2	0.714	0.733		0.91 (0.81 – 1.02)	0.1105			
rs16862964	3	LPP	G	1	0.480	0.262	0.323	2.59 (1.71 – 3.94)	6.7 × 10^−6^	0.97 (0.88 – 1.08)	0.5845	1.0000
				2	0.347	0.369		0.91 (0.82 – 1.01)	0.0796			
rs4834621	4		G	1	0.293	0.124	0.208	2.93 (1.75 – 4.93)	3.0 × 10^−5^	0.97 (0.86 – 1.09)	0.6395	1.0000
				2	0.216	0.233		0.91 (0.8 – 1.03)	0.1238			
rs7665789	4		A	1	0.894	0.743	0.798	2.92 (1.68 – 5.07)	8.9 × 10^−5^	0.99 (0.86 – 1.13)	0.8508	1.0000
				2	0.829	0.842		0.91 (0.79 – 1.05)	0.1975			
rs6595551	5	ZNF608	G	1	0.748	0.530	0.584	2.63 (1.72 – 4.01)	5.9 × 10^−6^	1.12 (1.01 – 1.24)	0.0337	0.4836
				2	0.661	0.648		1.06 (0.95 – 1.18)	0.3159			
rs3130932	6	POU5F1	C	1	0.460	0.272	0.387	2.27 (1.5 – 3.45)	1.0 × 10^−4^	1.05 (0.94 – 1.16)	0.3990	0.9999
				2	0.370	0.372		0.99 (0.89 – 1.1)	0.8889			
rs846514	6	LRFN2	A	1	0.849	0.678	0.679	2.66 (1.63 – 4.33)	6.3 × 10^−5^	1.04 (0.93 – 1.17)	0.4618	0.9999
				2	0.736	0.739		0.99 (0.88 – 1.11)	0.8048			
rs987105	6	MUT	G	1	0.939	0.807	0.911	3.71 (1.88 – 7.32)	7.1 × 10^−5^	1.25 (1.06 – 1.47)	0.0075	0.1388
				2	0.899	0.885		1.15 (0.97 – 1.37)	0.0983			
rs1325076	6	FUT9	G	1	0.444	0.243	0.232	2.5 (1.63 – 3.83)	2.1 × 10^−5^	1.02 (0.92 – 1.14)	0.7021	1.0000
				2	0.314	0.323		0.96 (0.86 – 1.07)	0.4651			
rs1449675	6		A	1	0.965	0.837	0.893	5.33 (2.3 – 12.36)	2.0 × 10^−5^	1.29 (1.09 – 1.52)	0.0025	0.0503
				2	0.902	0.885		1.19 (1 – 1.41)	0.0439			
rs10762033	10	CTNNA3	G	1	0.566	0.366	0.476	2.25 (1.51 – 3.36)	6.4 × 10^−5^	1.07 (0.97 – 1.18)	0.2078	0.9897
				2	0.470	0.466		1.01 (0.91 – 1.12)	0.7964			
rs4245124	11	SPATA19	C	1	0.697	0.485	0.565	2.44 (1.62 – 3.68)	1.7 × 10^−5^	1.07 (0.96 – 1.18)	0.2213	0.9922
				2	0.380	0.382		1.01 (0.9 – 1.12)	0.9090			
rs1413119	13		G	1	0.384	0.203	0.262	2.45 (1.57 – 3.82)	7.0 × 10^−5^	1.17 (1.05 – 1.31)	0.0050	0.0944
				2	0.304	0.282		1.11 (0.99 – 1.25)	0.0651			
**rs1408888**	**13**	**DACH1**	A	1	0.818	0.644	0.768	**2.49 (1.57 – 3.96)**	**8.4 × 10^−5^**	**1.21 (1.08 – 1.35)**	**9.1 × 10^−4^**	**0.0176**
				2	0.749	0.721		**1.15 (1.03 – 1.29)**	**0.0164**			
rs11069344	13	DOCK9	G	1	0.318	0.139	0.244	2.9 (1.76 – 4.78)	1.8 × 10^−5^	1.05 (0.92 – 1.18)	0.4860	0.9999
				2	0.196	0.201		0.97 (0.85 – 1.1)	0.6484			
rs11650227	17	MSI2	G	1	0.636	0.436	0.577	2.27 (1.52 – 3.39)	5.7 × 10^−5^	0.98 (0.88 – 1.08)	0.6715	1.0000
				2	0.546	0.566		0.92 (0.83 – 1.02)	0.1328			
rs13043334	20	CEBPB	C	1	0.626	0.421	0.47	2.31 (1.54 – 3.44)	3.9 × 10^−5^	1.01 (0.92 – 1.12)	0.7849	1.0000
				2	0.518	0.528		0.96 (0.87 – 1.06)	0.4301			
rs11089263	22	CESK1	C	1	0.717	0.525	0.702	2.3 (1.52 – 3.48)	7.4 × 10^−5^	1.09 (0.98 – 1.21)	0.1177	0.9099
				2	0.646	0.639		1.03 (0.93 – 1.15)	0.5715			

* **Nearest Entrez genes within 250 Kb.**

Stage 1 (genome scan) included 99 young-onset familial T2D patients and 101 controls. Stage 2 (replication stage) included 1468 T2D patients and 1485 controls. *P*_Allelic_ and *P*_permutation_ represent *P* values of allelic test and after permutation of 10,000 times based on 19 SNPs in stage 2, respectively.

Risk allele refers to the allele with a higher frequency in T2D patients than in controls in stage 1.

RAF (T2D) and RAF (Controls), risk allele frequencies in T2D patients and controls, respectively.

OR, odds ratio are reported with respect to the risk allele.

**Table 2:** Meta-analysis of risk association of *DACH1* rs1408888 with Type 2 diabetes in independent multi-ethnic Asian case-control cohorts.

	*n*			Risk allele frequency				
Study	T2D	Control	Total	T2D	Control	Weight	OR (95% CI)	*P*
Hong Kong Chinese	1567	1586	3153	0.753	0.716	21.9%	1.21 (1.08 – 1.35)	9.1×10^−4^
Shanghai Chinese	1779	1833	3612	0.763	0.761	24.57%	1.01 (0.91 – 1.12)	0.8504
Korean	749	616	1365	0.560	0.596	8.05%	0.96 (0.80 – 1.15)	0.6577
Singapore Chinese	2010	1945	3955	0.762	0.747	24.07%	1.09 (0.98 – 1.21)	0.1058
Singapore Malay	794	1240	2034	0.673	0.673	12.94%	0.98 (0.86 – 1.13)	0.7810
Japanese	471	582	1053	0.666	0.647	8.46%	1.09 (0.91 – 1.30)	0.3377
**Asian meta-analysis**	7370	7802	15172	--	--		1.07 (1.02 – 1.12)	**0.0112**
Heterogeneity test								0.1070

### Associations with quantitative traits in healthy adults

In 599 healthy adults and after adjustment for age and gender, systolic blood pressure (BP), Homeostasis Model Assessment index for insulin resistance (HOMA-IR) and β cell function (HOMA-β) and fasting insulin were associated with increasing number of alleles. Using multivariate analysis, the T-allele of *DACH1* rs1408888 was associated with systolic BP [β = 1.56(1.02–2.10) per T-allele, 0.61% variance explained], fasting plasma insulin [β = 0.072(–0.006–0.151) per T-allele, 1.05% variance explained] and HOMA-IR [β = 0.067 (–0.012–0.145) per T-allele, 0.97% variance explained] (Table 3).

**Table 3:** Clinical and metabolic characteristics of Hong Kong Chinese healthy adults stratified according to the genotypes of *DACH1* rs1408888.

Characteristics	GG (N = 55)	TG (N = 246)	TT (N = 298)	*P*
Body mass index (kg/m^2^)	22.7±3.8	22.9±3.3	23.0±3.3	0.801
Waist circumference (cm)	74.7±10.7	76.7±9.8	77.2±9.1	0.780
Hip circumference (cm)	92.8±6.4	93.3±6.3	93.6±5.8	0.624
**Systolic BP (mmHg)**	**111±14.5**	**114.3±16.1**	**116.8±16.9**	**0.030**
Diastolic BP (mmHg)	68.6±10.8	72.2±11	72.9±11.5	0.073
**Total cholesterol (mmol/l)**	**4.7±0.8**	**5±0.9**	**5.1±1**	**0.025**
Triglyceride (mmol/l)	0.7 (0.6 – 1.1)	0.9 (0.6 – 1.3)	0.9 (0.7 – 1.3)	0.547
HDL-C (mmol/l)	1.5±0.4	1.6±0.4	1.5±0.4	0.306
LDL-C (mmol/l)	2.8±0.8	3±0.8	3±0.9	0.071
**Fasting plasma glucose (mmol/l)**	**4.9 (4.5 – 5.2)**	**4.8 (4.6 – 5.1)**	**4.8 (4.6 – 5.1)**	**0.709**
**Fasting plasma insulin (pmol/l)**	**39.9 (23.1 – 51.2)**	**40.2 (24.4 – 55.9)**	**42.4 (29.7 – 61.6)**	**0.014**
**HOMA -IR**	**1.4 (0.8 – 1.9)**	**1.5 (0.9 – 2.0)**	**1.5 (1 – 2.2)**	**0.019**
HOMA-β	93.6 (68.1 – 135.6)	99 (63.4 – 162.6)	112.5 (71.0 – 167.6)	0.010
Insulinogenic index (mU/mmol):	13.9 (8.1 – 21.4)	15.5 (9.3 – 23.6)	16.3 (9.7 – 25.6)	0.2369
β Cell function (×10^−6^):	26.6 (19.4 – 35.2)	28.3 (18.6 – 38.7)	32.1 (21 – 44.2)	0.0804

Data are expressed as *n*, mean±SD or median (interquartile range). *P* values were calculated from linear regression adjusted for sex and age assuming an additive model. Homeostasis model assessment of insulin resistance (HOMA-IR) was calculated as (FPI ×FPG)÷22.5, and homeostasis model assessment of beta-cell function (HOMA-β) was calculated as FPI×20÷(FPG–3.5) where FPI = fasting plasma insulin and FPG = fasting plasma glucose. Beta cell function (×10^−6^) was determined by the formula: [insulin AUC_30min_ (min.pmol/l)÷glucose AUC_30min_ (min.mmol/l)] where AUC = area under curve. Insulinogenic index was calculated as (FPI during OGTT for 30 min – 0 min) ÷(FPG during OGTT for 30 min – 0 min) where OGTT = 75 gram oral glucose tolerance test.

### Clinical and pathological association with cardiovascular disease (CVD)

In the matched case-control cohort, Chinese T2D patients with CVD were more obese and had worse dyslipidaemia and renal function than those without CVD (Table 4). The TT/TG genotype was associated with an OR of 1.51 (*P* = 0.02) which remained significant after adjusting for estimated glomerular filtration rate (eGFR) with an OR of 1.54 (1.07–2.22 *P* = 0.019). In an autopsy series of 173 non-diabetic cases, rs1408888 did not depart from Hardy-Weinberg Equilibrium (HWE). Compared to cases with TG/GG genotype (n = 83, mean±SD age: 70.6±15.7 years, 43% female), cases with TT genotype (n = 90, age: 67.0±15.7 years, 38% female) were more likely to have a history of coronary heart disease (CHD) (17.8% versus 7.2%, *P* = 0.0375) and coronary arteriosclerosis (16.7% versus 6.0%, *P* = 0.0287). The respective ORs were 3.31(1.19–9.19, *P* = 0.0214) and 3.27(1.25–11.07, *P* = 0.0184) after adjustment for age and sex.

**Table 4:** Clinical and metabolic characteristics of a case-control cohort of Hong Kong Chinese Type 2 diabetic patients with or without cardiovascular disease (CVD) matched for age, sex and disease duration.

	No CVD	CVD		*P* values	
N	953	953			
Sex (% of male)	48.3%	48.3%			
Age (years)	64.1±10.3	64.1±10.3			
Age of diagnosis (years)	54.3±12.7	54.4±12.7			
Diabetes duration (years)	9.4±6.7	9.4±6.7			
Body mass index (kg/m^2^)	24.4±4.6	24.7±4.9		0.124	
Waist circumference (cm)	85.2±11.5	86.3±12.8		0.063	
Systolic blood pressure (mmHg)	140.0±22.8	143.3±21.9		<0.001	
Diastolic blood pressure (mmHg)	76.0±12.2	76.9±11.7		0.110	
Glycated hemoglobin (%)	7.4(6.4–8.6)	7.8(6.8–9.3)		<0.001	
High density lipoprotein cholesterol (mmol/L)	1.27(1.04–1.51)	1.17(1.0–1.4)		0.004	
Low density lipoprotein cholesterol (mmol/L)	3.20(2.6–3.8)	3.30(2.7–4.0)		0.050	
Triglyceride (mmol/L)	1.37(0.93–2.0)	1.52(1.05–2.23)		0.053	
Urinary albumin:creatinine ratio (mg/mmol)	2.72(0.91–14.3)	7.5(1.80–49.3)		<0.001	
Estimated GFR (ml/min/1.73 m^2^)	97.9(77.5–116.8)	89.1(65.7–109.1)		<0.001	
*DACH1* rs1408888			Model	OR(95%CI); *P* values	^*^OR(95%CI); *P* values
TT count (%)	522(54.8)	524(55.0)	Dominant	**1.51(1.06–2.17); 0.024**	**1.54(1.07–2.22); 0.019**
TG count (%)	353(37.0)	376(39.5)	Recessive	1.01(0.84–1.21); 0.927	1.04(0.86–1.24); 0.709
GG count (%)	78(8.2)	53(5.5)	Allelic	1.08(0.93–1.24); 0.319	1.10(0.95–1.27); 0.209
Types of CVD (number, %)					
Coronary heart disease	-	541, 56.8%			
Stroke	-	453, 47.5%			
Peripheral vascular disease	-	223, 23.4%			
1 vascular bed	-	712, 74.7%			
2 vascular beds	-	218, 22.9%			
3 vascular beds	-	23, 2.4%			

Data are expressed in mean±SD or median(interquatile range) or n, %). ^*^ *P* values and ORs were estimated by the logistic regression with adjustment for logarithm of eGFR.

Cardiovascular diseases was diagnosed based on clinical history and assessment at enrolment to the Hong Kong Diabetes Registry and/or subsequent events defined by the International Classification of Diseases, Ninth Revision (ICD-9), retrieved from the Hong Kong Death Registry and Hong Kong Hospital Authority (HA) Central Computer System. Coronary heart disease (CHD) was defined as myocardial infarction (ICD-9 code 410), ischemic heart disease (ICD-9 code 411-414) or death due to CHD (ICD-9 code 410-414). Stroke was defined as non-fatal (ICD-9 code 432-434, 436) or fatal ischemic stroke (ICD-9 code 432-438), or, hemorrhagic stroke as defined by fatal and non-fatal subarachnoid hemorrhage (ICD-9 code 430), intracerebral hemorrhage (ICD-9 code 431) or other/unspecified intracranial hemorrhage (ICD-9 code 432). Peripheral vascular disease (PVD) was defined as ankle-brachial ratio<0.9 using Doppler ultrasound scan, diabetes with peripheral circulatory disorders (ICD-9 code 250.7), gangrene (ICD-9 code 785.4), angiopathy in diseases classified elsewhere (ICD-9 code 443.81), peripheral vascular disease unspecified (ICD-9 code 443.9), other peripheral vascular shunt or bypass (procedure code 39.29), insertion of non-drug-eluting peripheral vessel stents (procedure code 39.90) or amputation of lower limb (procedure code 84.1) without a traumatic amputation diagnosis code (ICD-9 code 895-897).

### Bioinformatics analysis and expression study

**Figure 2:**
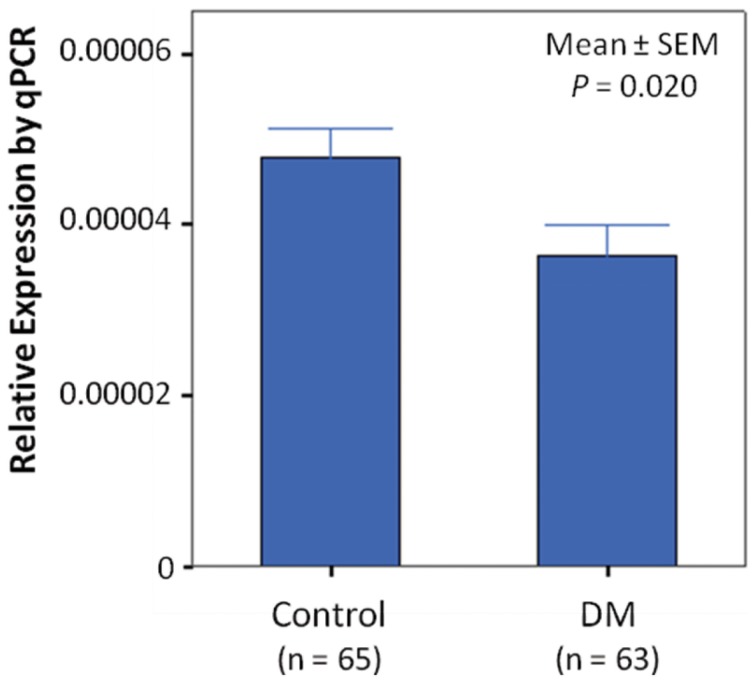
Expression of *DACH1* detected by quantitative real-time PCR, in peripheral blood mononuclear cells (PBMC) extracted from 65 control subjects and 63 young-onset type 2 diabetic patients (DM). Expression level was normalized to the expression of β actin using the ΔΔCt method. The results are represented as mean ± standard error of the mean (SEM) with age and sex adjustment.

Two neighboring SNPs in weak linkage disequilibrium (LD) (r^2^≈0.5) with rs1408888 (rs9572813 and rs17791181) also showed nominal association with T2D (*P* = 0.01–0.001) in the GWAS analysis (Figure 1). Bioinformatics analysis revealed that the region between rs1408888 and rs9572813 overlapped with a regulatory element conserved from fugu fish to human [15]. On datamining, this element [OREG0002711 (http://www.oreganno.org/oregano/) or chr13:72,425,787-72,428,335 (hg19) (http://enhancer.lbl.gov/frnt_page_n.shtml)] shows an enhancer activity which directs the distinct expression of a β-galactosidase reporter gene in the eye, cranial nerve, forebrain, hindbrain and neural tube in the mouse embryos [15], [16]. In this region, another non-coding element (*CNE803*) [17], highly conserved in vertebrates, shows homology to an EST from the human eye (BY797940) (Figure 1). We sequenced the region between rs1408888 and rs9572813 in 74 controls and 82 cases who had GWAS data and did not discover novel variants in the *CNE803* nor in the surrounding regions. In this genomic region containing multiple SNPs, 3 SNPs (rs17252745, rs17252752 and rs57143718) with marked inter-ethnic differences in the NCBI SNP database (Figure 1) (http://www.ncbi.nlm.nih.gov/snp/) [minor allele frequency (MAF)<0.05 in CEU (Caucasians) versus 0.4–0.5 in CHB/JBT (Asians)] showed nominal association with YOD with OR ranging from 1.36 to 1.53. They were further genotyped in 206 controls and 390 subjects with YOD with one of the SNPs (T allele of rs57143718) showing association in the combined cohort of 472 cases and 280 controls [OR = 1.26(1.02–1.56), *P* = 0.036]. We used reverse transcription PCR and Northern blot analysis to examine the expression of *CNE803* in PBMC, pancreatic progenitor cells (PPC) [18], HCT116 and HKCI-2 cancer cells which was negative. Expression of *DACH1* was detected in PBMC and PPC. Using quantitative real-time PCR, lower *DACH1* expression was found in PBMC from patients with YOD compared to control subjects (*P* = 0.02 for regression with age and sex adjustment, *P* = 0.005 for Wilcoxon rank sum test) (Figure 2).

## Discussion

In an adequately powered case-control cohort of familial YOD characterized by obesity, we discovered several common SNPs (allele frequency>0.3) with ORs of 2–2.5, including the T allele of rs1408888 of *DACH1* with allele frequency of 0.75 and an OR of 2.49. In a subset of this cohort, we found reduced expression of *DACH1* in PBMC in patients with YOD. In the stage 2 experiment, we successfully genotyped 19 SNPs with the lowest *P* value and replicated the association of rs1408888 with an OR of 1.21 and a global *P*<0.05 in a random case-control cohort of 2953 Chinese with older age of diagnosis. This was followed by confirmation in 15,172 Asian subjects with an OR of 1.07. We further demonstrated that the T allele was associated with insulin resistance and high BP in normal subjects, CVD in patients with T2D, and coronary arteriosclerosis in autopsy samples. Bioinformatics analysis revealed its location in a conserved region within the intron of *DACH1,* implicated in pancreatic islet development [13]. Unlike the novel SNP rs10229583 at 7q32 near *PAX4* discovered in a meta-analysis of 3 GWAS comprising 684 T2D patients and 955 controls of Southern Han Chinese descent with confirmation in a multi-ethnic population (*P*_meta_ = 2.3×10^−10^ in East Asians; *P*  = 8.6×10^−3^ in Caucasians) [12], the risk association of rs1408888 with T2D was increasingly attenuated in a multi-ethnic population, suggesting that this SNP might be more relevant to Asians undergoing rapid transition characterized by obesity and young age of diagnosis [6].

### Known function of *DACH1*

*DACH1,* located on chromosome 13q21, is the mammalian homologue of the *Drosophila* dachshund (*Dac)* gene. It encodes a well-conserved nuclear protein capable of binding to DNA and has two highly conserved domains (*DachBox-N and DachBox-C*) from *Drosophila* to humans [19], [20]. It is a key component of the retinal determination gene network that governs cell fate and plays a key role in ocular, limb, brain and gonadal development [21]. *DACH1* knockout mice die shortly after birth, with no gross histological abnormalities observed in eyes, limbs, or brain tissues, suggesting its possible role in perinatal development [22]. Herein, *DACH1* is a critical transcription factor in tissue differentiation and organ development. One of its gene targets is the mothers against decapentaplegic homolog 4 (*SMAD4*) which upon binding with *DACH1* can result in repression of TGFβ signaling and TGFβ-induced apoptosis [23]. In this regard, increased TGFβ1 activity has been implicated in heart and vascular development, hypertension and progressive myocardial fibrosis [24], [25]. In a recent proof-of-concept analysis, researchers merged co-expression and interaction networks and detected/inferred novel networks to explain the frequent but not invariable coexistence of diabetes and other dysfunctions. One of these networks included regulation of *TGFBRII* which facilitated oxidative stress with expression of early transcription genes via *MAPK* pathway leading to cardiovascular-renal complications. The second network proposed the interaction of beta-catenin with *CDH5* and *TGFBR1* through *Smad* molecules to contribute to endothelial dysfunction [26], often a precursor of CVD [27].

### *DACH1*, T2D and Cardiovascular-renal disease

In the Wellcome Trust Case Control Consortium [28], Diabetes Genetics Initiative [29] and a recent Chinese GWAS [3], *DACH1* was among the list of genes with nominal association (*P*<0.05) with T2D. In the Emerging Risk Factor Collaboration Study, diabetes was associated with multiple morbidities including cardiovascular and renal disease [30]. In a recent meta-analysis of GWAS data in 67,093 individuals of European ancestry, *DACH1* was one of the susceptibility loci for reduced renal function [31]. Although we did not find association between rs1408888 and renal function in our study, these consistent findings highlight the possible role of *DACH1* in regulating functions of multiple organs.

In support of these genetic associations, in a mouse model of diet-induced β-cell dysfunction, islet *DACH1* gene expression was reduced in prediabetic animals fed a high-fat diet [32]. In both zebrafish and mice, loss of *DACH1* resulted in reduced numbers of all islet cell types, including β-cells [13]. Although deletion of *DACH1* in mice did not affect the number of PPC, it blocked the perinatal burst of proliferation of differentiated β-cells [13]. In *Drosophila*, there was strong expression of *Dac,* the homolog of *DACH1/2*, in insulin-producing cells with *Dac* interacting physically with *Pax6* homolog Eyeless (*Ey*) to promote expression of insulin-like peptides. In a similar vein, the mammalian homolog of *Dac, DACH1/2*, also facilitated the promoting action of *Pax6* on the expression of islet hormone genes in cultured mammalian cells [14].

Given the strong links between T2D and CVD, the association of TT/TG genotype of *DACH1* with CVD with an odds ratio of 1.54, after adjustment for age, sex, disease duration and eGFR was noteworthy. Patients with CVD were more obese and had more risk factors including high BP. Interestingly, in normal subjects, the T allele was linearly associated with BP and insulin levels which are well known risk factors for CVD [11]. In the autopsy series, TT carriers had 2–3 fold increased risk of coronary arteriosclerosis and CHD. These consistent findings in independent cohorts at different stages of the spectrum of cardio-metabolic disease, together with experimental studies from other groups, strongly support the role of *DACH1* in these complex diseases.

The complexity of human evolution and natural selection by external forces, including but not limited to temperature, foods, infections, can result in diversity of genomic architecture and expression, making replication of genetic association of complex diseases challenging [33]. Given the rapid westernization of Hong Kong Chinese within less than a century, we hypothesize that *DACH1* may be a thrifty gene which regulates growth to improve survival chances during time of hardship but increases risk of obesity, prediabetes, YOD and CVD during time of affluence [34]. Hitherto, apart from maturity onset diabetes of the young [35], the genetics of familial YOD characterized by obesity have not been well studied and these results might motivate further research in subjects with these phenotypes to confirm or refute our hypothesis.

### Possible significance of rs1408888 of *DACH1*

The risk allele rs1408888 is located in the first intron of *DACH1* within the vicinity of conserved elements [16], which can direct a unique gene expression pattern resembling the embryonic expression pattern of *DACH1* [22]. One of these elements, *CNE803* located 1.6 Kb from rs1408888 (Figure 1), showed sequence homology to an EST from an eye library (BY797940). We sequenced this region in the original GWAS cohort but did not find any novel SNPs. Three SNPs in this region which were common in Chinese but rare in Caucasians showed nominal associations with T2D in the discovery cohort, with rs57143718 replicated in an expanded case-control cohort of YOD. Using PPC, we were unable to detect expression of *CNE803* but found multiple *DACH1* isoforms (data not shown). On bioinformatics analysis, rs1408888 is located in a region with multiple consensus transcription factor binding sites and closely associated with open and active chromatins (Table S1), suggesting that this region may regulate *DACH1* expression. In support of these predictions, we found reduced expression of *DACH1* in PBMC in patients with YOD. Although there is no direct link between rs1408888 and rs57143718 to the expression level of *DACH1* in PBMC, the reduced expression of *DACH1* in YOD patients supports importance of *DACH1* and agrees with the bioinformatic analysis of the region surrounding rs1408888. The functional significance of this SNP/locus requires further exploration.

### Limitations and conclusion

In this multi-staged experiment, we have discovered risk association of an intronic SNP (rs1408888) of *DACH1* with YOD, BP, insulin resistance and CVD in Chinese populations. Although no significant association between rs1408888 and the YOD subgroup in the stage 1 replication cohort was found (*P*>0.1), possibly due to small sample size, the age of diagnosis was relatively young in our discovery (31.8±7.7 years) and replication cohorts for T2D (44.0±13.6 years) and that for CVD (54.3±12.7 years) (Tables 4 and 5) compared to most GWAS. Given that these findings were mainly found in Asian population where YOD is a predominant feature, our findings highlight the need for more genetic research in YOD. Together with the known function of *DACH1* on developmental biology and regulation of insulin secretion and its reduced expression in human PBMC associated with YOD, these findings add to the growing body of knowledge regarding the candidacy role of *DACH1* for cardio-metabolic dysfunction, which may manifest as YOD in populations undergoing rapid transition in nutrition and lifestyles.

**Table 5:** Clinical profiles of discovery, replication and validation cohorts in a 3-stage genome wide association study in Asian populations.

	Hong Kong Chinese						Shanghai Chinese		Japanese		Korean		Singapore Chinese (Illumina 610quad)		Singapore Chinese (Illumina1 Mduov3)		Singapore Malay	
	Discovery cohort		Replication cohort															
	**T2D**	**Controls**	**T2D**	**Controls Adult**	**Controls adolescents**		**T2D**	**Controls**	**T2D**	**Controls**	**T2D**	**Controls**	**T2D**	**Controls**	**T2D**	**Controls**	**T2D**	**Controls**
n	99	101	1468	507	978		1892	1808	471	582	761	632	1082	1006	928	939	794	1240
Male	40	37	592	234	457		988	749	262	204	354	286	402	217	602	599	405	645
Age (years)	40.6±8.8	37.4±10.1	50±13.8	42.2±10.4	15.3±1.9		61.2±12.6	57.3±12.3	61.6±10.4	67.9±9.1	59.2±9.9	64.7±3.6	-	47.7±11.1	-	46.7±10.2	62.3±9.9	56.9±11.4
Age-at-diagnosis (year)	31.8±7.7	--	44.0±13.6	--	--		54.1±11.8	-	46.2±8.0	-	50.0±10.1	-	55.7±12.0	-	52.2±14.4	-	-	-
Body mass index (kg/m^2^)	30.9±4.4	20.8±2	24.8±3.9	23.3±3.4	19.9±3.6		24.1±3.5	23.6±4.2	24.2±3.8	22.4±3.2	24.5±2.9	23.5±3.1	25.3±3.9	22.3±3.7	25.4±3.8	22.8±3.4	27.8±4.9	25.1±4.8
HbA_1C_ (%)	8.0±1.9	--	8.0±2.0	--	--		9.2±2.4	-	7.9±1.6	5.0±0.4	8.1±1.6	5.3±0.3	-	-	-	-	8.1±1.8	5.6±0.3
Fasting plasma glucose (mmol/l)		4.7±0.4	--	4.9±0.4	4.7±0.3		13.0±5.2	5.0±0.5	-	-	8.6±2.6	5.0±0.5	-	4.7±0.45	-	4.7±0.5	-	-

## Methods

### Risk association with diabetes using multiple cohorts

The clinical characteristics of the discovery and replication cohorts of the Asian population as well as methods of genotyping and genetic analysis have been reported [12]. In brief, the discovery cohort (stage 1) consisted of 99 obese Chinese subjects (BMI≥27 kg/m^2^ or waist≥90 cm in men or ≥80 cm in women) with YOD (age of diagnosis<40 years) and at least one affected first-degree relative selected from the HKFDS [8], [9] and the Hong Kong Diabetes Registry (HKDR) [36]. The 101 age- and sex-matched control subjects were selected from a community-based health promotion program [37]. The replication cohort (stage 2) consisted of 1468 T2D subjects selected from the HKDR [36] and unrelated subjects from the HKFDS [8] while the control cohort consisted of 507 healthy volunteers [37] and 978 adolescents [38]. The validation cohorts (stage 3) consisted of 1892 cases and 1808 controls from Shanghai [39]; 749 cases and 616 controls from Korea [40]; 2804 cases (2010 Chinese, 794 Malay) and 2185 controls (1945 Chinese, 1240 Malays) from Singapore [41] and 471 cases and 582 controls from Japan [40] (Table 5). Written informed consent was obtained from all participants or their parents with approval by the Clinical Research Ethics Committee of the Chinese University of Hong Kong, the ethics committee of the Wakayama Medical University, the institutional review boards of the Clinical Research Institute in the Korea Seoul National University Hospital and the Shanghai Jiao Tong University Affiliated Sixth People's Hospital.

### Quantitative traits, CVD and clinico-pathological features

**Figure 3:**
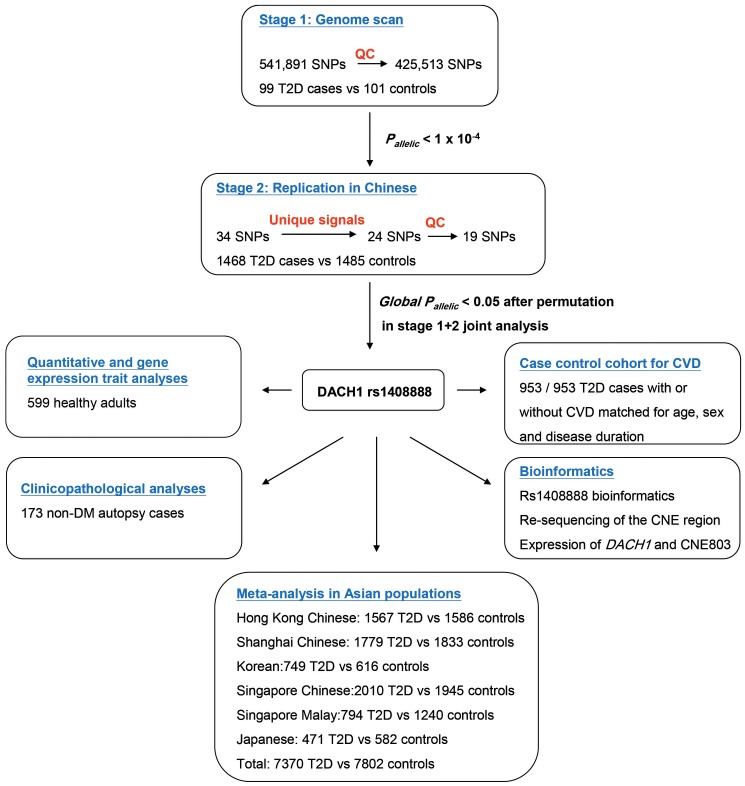
Flow chart summarizing the study design, subject recruitment, experiments and data analysis.

All subjects in the Hong Kong control cohort had documentation of anthropometric indexes, BP, cardiovascular risk factors, plasma insulin and glucose during 75 gram oral glucose tolerance test [42]. The case-control cohort was selected from the HKDR set up in 1995 as part of a quality improvement program using structured protocols [36]. Using this cohort, we selected a case-control cohort of CVD (953/953) matched for age, sex and disease duration. Definitions of CVD (including ischaemic heart disease, stroke and peripheral vascular diseases) were based on clinical assessments and the International Classification of Disease 9^th^ version. In an autopsy series with documentation of pathological features and clinical history [43], [44], we extracted genomic DNA from archived paraffin blocks using white blood cell-concentrated spleen tissues for genotyping. Figure 3 summarizes the selection criteria and study flow.

### Genotyping

**Discovery cohort.** The discovery cohort (99 cases and 101 controls) were assayed with Illumina HumanHap550-Duo BeadChip at deCODE Genetics. Of the 541,891 genotyped autosomal SNPs, 116,378 (21%) SNPs were excluded due to call rate<0.95 (n = 2311); MAF<0.05 (n = 113,596) and significant departure from HWE in control subjects (*P*<0.001) (n = 947), giving 425,513 SNPs on chromosome 1–22 for final analysis after checking for population stratification.

**Replication cohort.** In the second stage, 24 SNPs with the lowest *P* value (*P*<1×10^−4^ in allelic test) were genotyped in the replication cohort. Only one SNP was genotyped for locus with multiple SNPs in high LD (r^2^>0.6). Genotyping was performed at the McGill University and Genome Quebec Innovation Centre using primer extension of multiplex products with detection by MALDI-TOF mass spectroscopy on a Sequenom MassARRAY platform (San Diego, CA, USA). Of 24 genotyped SNPs, 5 were excluded due to low call rate (<90%). All remaining 19 SNPs were in HWE in controls with a concordance rate of 96% in 65 blinded duplicate samples.

**Validation cohorts.** rs1408888 which showed significance in stage 1, 2 and combined cohorts were genotyped in the Asian populations using the following methods: 1) Shanghai Chinese: MassARRAY platform (MassARRAY Compact Analyzer, Sequenom, San Diego, CA, USA) with 97.5% call rate and 100% concordance rate; 2) Korea: Assay-on-Demand TaqMan assays (Applied Biosystems, Foster City, CA, USA) and ABI PRISM 7900HT Sequence Detection System (Applied Biosystems, Foster City, CA, USA) with 99.4% call rate and 100% concordance rate based on 13 duplicates; 3) Japan: TaqMan SNP genotyping system (Applied Biosystems, Foster City, CA) and ABI PRISM 7700 system with 20% of samples directly sequenced using Sanger sequencing and analyzed with an ABI 3100 capillary sequencer with 100% concordance rate. For the Singapore study, the Chinese samples were genotyped on the 610Quad and 1Mduov3 platforms while the Malay samples were genotyped on the Illumina HumanHap 6100Quad. We removed SNPs with call rate <95%, or departure from HWE (*P* <0.0001), or which were monomorphic.

**Autopsy series.** DNA was obtained using a modified DNA-extraction protocol [43] for genotyping using a Taqman kit from ABI and an ABI 7900HT Fast Real-Time PCR System.

### Re-sequencing of the rs1408888 genomic region

The genomic region between rs1408888 and rs9572813 was PCR amplified in 2 DNA fragments for sequencing. The fragment close to rs1408888 was amplified by *DACH1-*F (5′-TCTTGCTATAAAATGCATGAAAGGAG-3′) and 1R (5′-ATAGCCAAAGGGAGGGAAAA-3′). The 1.7 Kb DNA fragment was sequenced by primers 1F (5′-AAGGGCCCATGACAGGAATG-3′) and 3F (5′-TCACTCAAGATGAGTTCACACCA-3′) in one direction and 2R (5′-GTTATTATCGGCCCAATTCC-3′) in opposite direction. The primer 1F covers rs57143718 and the primer 3F covers *CNE803*. The fragment close to rs9572813 was amplified by *CNE803*-1F (5′-TAATACCATTGCCCCAAGGA-3′) and *DACH1*-R (5′-CAGCAAATCCCAGCGTAGCAC-3′). The fragment was sequenced by *CNE803*-2F (5′-TGACCCAGCTCTCATCCTTT-3′) to cover rs17252745 and rs17252752. The DNA sequencing data were deposited to the NCBI Trace Archive (http://www.ncbi.nlm.nih.gov/Traces/trace.cgi?&cmd=retrieve&val=CENTER_NAME%20%3D%20%22CUHK%22&dopt=info&size=330&dispmax=1&page=1&seeas=Show), with the TI number 2335839769-2335840098 (330 traces).

### Detection of *DACH1* and CNEs expression by reverse-transcription PCR

Expression of *CNE803* and *DACH1* transcripts in various cell types were detected by RT-PCR. Expression of the *CNE* (220bp) was detected by primers 5′-TAATACCATTGCCCCAAGGA-3′ and 5′-TTTGGATTTCAGCCTTGTCA-3′. Expression of *DACH1* was detected using 5′-CTGCACCAACGCAAGTTCTA-3′ and 5′-ATAAGCCCATCAGCATCTGG-3′ as primers. Expression of β actin was used as a positive control using 5′-AGAGCTACGAGCTGCCTGAC-3′ and 5′-AGCACTGTGTTGGCGTACAG-3′ as primers. Expression of *DACH1* in PBMC was quantified by real-time PCR using the SYBR Green method with 5′-GTGGAAAACACCCCTCAGAA-3′ and 5′-CGAAGTCCTTCCTGGAGATG-3′ as primers in an ABI 7900HT Real-Time PCR system. Expression level was normalized to the expression of β actin for comparison using the ΔΔCt method. The result was analyzed by Wilcoxon rank sum test and regression analysis with sex and age adjustment.

### Statistical analysis

We used PLINK v1.07 (http://pngu.mgh.harvard.edu/purcell/plink/), Statistical Analysis Software v.9.1 (SAS Institute, Cary, NC, USA) or Statistical Package for Social Sciences for Windows v.15 (SPSS, Chicago, IL, USA) for all statistical analyses, unless specified otherwise. All data are presented as mean±SD or median (interquartile range) unless specified. Categorical variables were compared using χ^2^ test, Fisher’s exact test and logistic regression, expressed as ORs and 95% CI as appropriate. In healthy controls, fasting plasma insulin, HOMA-IR and HOMA-β were logarithmically transformed due to skewed distributions. Genotype-phenotype associations were tested by multivariable linear regression adjusted for sex and age under the additive genetic model expressed in β coefficients with 95%CI. Between-group comparisons were performed by χ^2^ test, Student’s t-test or Wilcoxon Rank Sum as appropriate. We used logistic regression to examine genetic association with CVD in a case-control cohort matched for age, sex and disease duration with further adjustment for logarithm of eGFR, expressed as OR (95%CI). A two-tailed *P* value*<*0.05 was considered significant.

### Sample size estimation

Assuming an additive model with allele frequencies of 0.05–0.30, and an OR of 1.2–3.0 (for a prevalence of 0.1), we used the Genetic Power Calculator [45] to estimate the power for stage 1 (genome scan) and stage 2 (replication) at α levels of 1×10^−4^ and 0.05, respectively. For allele frequency>0.2, a sample size of 200 had 90% power to detect an OR of 3 and 75% power for an OR of 2.5. For the replication cohort with a sample size of 3000, we had 90% power to confirm an OR 1.2 for allele frequency>0.2. For the risk association with CVD, for SNP with allele frequency>0.2, a sample size of 2000 had over 90% power to confirm an OR of 1.5.

### GWAS and meta-analysis

Distributions of all genotypes were analyzed for deviation from HWE by χ^2^ test with one degree of freedom. In stage 1 experiment, we estimated possible familial relationship using estimates of identity-by-descent (IBD) derived from pair-wise analyses of 102,919 independent (r^2^≈0) and quality SNPs. We did not detect population stratification using multidimensional scaling analysis and the inflation factor λ for GC. GC [46] was applied to correct for relatedness of the subjects and adjust for potential population stratification. The inflation factor λ was estimated by taking the median of the distribution of the χ^2^ statistic from 425,513 quality SNPs in allelic test, and then divided by the median of the expected χ^2^ distribution. The Quantile-Quantile plots were used to compare the observed and expected distributions for the 1*df* χ^2^ statistics generated from allelic tests with or without correction for GC in the discovery stage. We calculated the corrected *P* values by dividing the observed χ^2^ statistic by λ. For the top signals taken forward for replication, we used Haploview v4.1 to generate pairwise LD measures and the Manhattan plot as well as LocusZoom v1.1 to generate the regional plots for the interested gene loci. For analysis of data from stage 1, 2 and combined dataset, we used allelic χ^2^ tests in 2×2 contingency tables to derive the OR after correction for multiple testings in 10,000 permutations. We used MIX v1.7 [47] to perform meta-analysis and calculated the combined estimates of ORs by weighting the natural log-transformed ORs (with respect to the same allele) of each study using the inverse of their variance under the fixed effect model. Cochran’s *Q* statistic (*P* <0.05) and *I* ^2^ were used to assess heterogeneity of ORs between studies.

## Supporting Information

Table S1 **Summary of bioinformatics analysis of rs1408888 of** ***DACH1*** **(Ch13:70910099-71339331) based on NCBI Build 36.**
(DOC)

## References

[1] McCarthy MI (2010) Genomics, type 2 diabetes, and obesity. N Engl J Med 363: 2339–2350. 21142536 10.1056/NEJMra0906948

[2] Cho YS, Chen CH, Hu C, Long J, Hee Ong RT, et al (2011) Meta-analysis of genome-wide association studies identifies eight new loci for type 2 diabetes in east Asians. Nat Genet 44: 67–72. 22158537 10.1038/ng.1019 PMC3582398

[3] Li H, Gan W, Lu L, Dong X, Han X, et al (2013) A Genome-Wide Association Study Identifies GRK5 and RASGRP1 as Type 2 Diabetes Loci in Chinese Hans. Diabetes 62: 291–298. 22961080 10.2337/db12-0454 PMC3526061

[4] Shu XO, Long J, Cai Q, Qi L, Xiang YB, et al. (2010) Identification of new genetic risk variants for type 2 diabetes. PLoS Genet 6. 10.1371/journal.pgen.1001127 PMC2940731 20862305

[5] Ng MCY, Lee SC, Ko GTC, Li JKY, So WY, et al (2001) Familial early onset type 2 diabetes in Chinese: the more significant roles of obesity and genetics than autoimmunity. Diabetes Care 24: 667–671. 10.2337/diacare.24.4.663 11315828

[6] Chan JC, Malik V, Jia W, Kadowaki T, Yajnik CS, et al (2009) Diabetes in Asia: epidemiology, risk factors, and pathophysiology. JAMA 301: 2129–2140. 19470990 10.1001/jama.2009.726

[7] Wang Y, Cai JH, Ma RCW, Song XY, Chan JCN, et al. (2013) Age of diagnosis, chronic hepatitis B viral infection and cardiovascular-renal endpoints in type 2 diabetes: a 10-year prospective cohort analysis by structural equation modeling. BMC Public Health: in press.

[8] Li JK, Ng MC, So WY, Chiu CK, Ozaki R, et al (2006) Phenotypic and genetic clustering of diabetes and metabolic syndrome in Chinese families with type 2 diabetes mellitus. Diabetes Metab Res Rev 22: 46–52. 16021651 10.1002/dmrr.577

[9] Ng MC, So WY, Lam VK, Cockram CS, Bell GI, et al (2004) Genome-wide scan for metabolic syndrome and related quantitative traits in Hong Kong Chinese and confirmation of a susceptibility locus on chromosome 1q21–q25. Diabetes 53: 2676–2683. 15448100 10.2337/diabetes.53.10.2676

[10] Despres JP, Lemieux I (2006) Abdominal obesity and metabolic syndrome. Nature 444: 881–887. 17167477 10.1038/nature05488

[11] Ford ES (2005) Risks for all-cause mortality, cardiovascular disease, and diabetes associated with the metabolic syndrome: a summary of the evidence. Diabetes Care 28: 1769–1778. 15983333 10.2337/diacare.28.7.1769

[12] Ma RC, Hui C, Tam CH, Zhang R, Kwan P, et al (2013) Genome-wide Association Study in Chinese Identifies a Susceptibility Locus for Type 2 Diabetes at 7q32 near PAX4. Diabetologia 56: 1291–1305. 23532257 10.1007/s00125-013-2874-4 PMC3648687

[13] Kalousova A, Mavropoulos A, Adams BA, Nekrep N, Li Z, et al (2010) Dachshund homologues play a conserved role in islet cell development. Dev Biol 348: 143–152. 20869363 10.1016/j.ydbio.2010.09.007 PMC2997432

[14] Okamoto N, Nishimori Y, Nishimura T (2012) Conserved role for the Dachshund protein with Drosophila Pax6 homolog Eyeless in insulin expression. Proc Natl Acad Sci U S A 109: 2406–2411. 22308399 10.1073/pnas.1116050109 PMC3289324

[15] Nobrega MA, Ovcharenko I, Afzal V, Rubin EM (2003) Scanning human gene deserts for long-range enhancers. Science 302: 413. 14563999 10.1126/science.1088328

[16] Pennacchio LA, Ahituv N, Moses AM, Prabhakar S, Nobrega MA, et al (2006) In vivo enhancer analysis of human conserved non-coding sequences. Nature 444: 499–502. 17086198 10.1038/nature05295

[17] Woolfe A, Goodson M, Goode DK, Snell P, McEwen GK, et al (2005) Highly conserved non-coding sequences are associated with vertebrate development. PLoS Biol 3: e7. 15630479 10.1371/journal.pbio.0030007 PMC526512

[18] Suen PM, Zou C, Zhang YA, Lau TK, Chan J, et al (2008) PDZ-domain containing-2 (PDZD2) is a novel factor that affects the growth and differentiation of human fetal pancreatic progenitor cells. Int J Biochem Cell Biol 40: 789–803. 18037333 10.1016/j.biocel.2007.10.020

[19] Wu K, Liu M, Li A, Donninger H, Rao M, et al (2007) Cell fate determination factor DACH1 inhibits c-Jun-induced contact-independent growth. Mol Biol Cell 18: 755–767. 17182846 10.1091/mbc.E06-09-0793 PMC1805093

[20] Zhou J, Wang C, Wang Z, Dampier W, Wu K, et al (2010) Attenuation of Forkhead signaling by the retinal determination factor DACH1. Proc Natl Acad Sci U S A 107: 6864–6869. 20351289 10.1073/pnas.1002746107 PMC2872468

[21] Popov VM, Wu K, Zhou J, Powell MJ, Mardon G, et al (2010) The Dachshund gene in development and hormone-responsive tumorigenesis. Trends Endocrinol Metab 21: 41–49. 19896866 10.1016/j.tem.2009.08.002 PMC2818438

[22] Davis RJ, Shen W, Sandler YI, Amoui M, Purcell P, et al (2001) Dach1 mutant mice bear no gross abnormalities in eye, limb, and brain development and exhibit postnatal lethality. Mol Cell Biol 21: 1484–1490. 11238885 10.1128/MCB.21.5.1484-1490.2001 PMC86694

[23] Wu K, Yang Y, Wang C, Davoli MA, D'Amico M, et al (2003) DACH1 inhibits transforming growth factor-beta signaling through binding Smad4. J Biol Chem 278: 51673–51684. 14525983 10.1074/jbc.M310021200

[24] Ramos-Mondragon R, Galindo CA, Avila G (2008) Role of TGF-beta on cardiac structural and electrical remodeling. Vasc Health Risk Manag 4: 1289–1300. 19337543 10.2147/vhrm.s3985 PMC2663446

[25] Yang SN, Burch ML, Tannock LR, Evanko S, Osman N, et al (2010) Transforming growth factor-beta regulation of proteoglycan synthesis in vascular smooth muscle: contribution to lipid binding and accelerated atherosclerosis in diabetes. J Diabetes 2: 233–242. 20923499 10.1111/j.1753-0407.2010.00089.x

[26] Sengupta U, Ukil S, Dimitrova N, Agrawal S (2009) Expression-based network biology identifies alteration in key regulatory pathways of type 2 diabetes and associated risk/complications. PLoS One 4: e8100. 19997558 10.1371/journal.pone.0008100 PMC2785475

[27] Libby P, Theroux P (2005) Pathophysiology of coronary artery disease. Circulation 111: 3481–3488. 15983262 10.1161/CIRCULATIONAHA.105.537878

[28] Zeggini E, Weedon MN, Lindgren CM, Frayling TM, Elliott KS, et al (2007) Replication of genome-wide association signals in UK samples reveals risk loci for type 2 diabetes. Science 316: 1336–1341. 17463249 10.1126/science.1142364 PMC3772310

[29] Saxena R, Voight BF, Lyssenko V, Burtt NP, de Bakker PI, et al (2007) Genome-wide association analysis identifies loci for type 2 diabetes and triglyceride levels. Science 316: 1331–1336. 17463246 10.1126/science.1142358

[30] Seshasai SR, Kaptoge S, Thompson A, Di Angelantonio E, Gao P, et al (2011) Diabetes mellitus, fasting glucose, and risk of cause-specific death. N Engl J Med 364: 829–841. 21366474 10.1056/NEJMoa1008862 PMC4109980

[31] Kottgen A, Pattaro C, Boger CA, Fuchsberger C, Olden M, et al (2010) New loci associated with kidney function and chronic kidney disease. Nat Genet 42: 376–384. 20383146 10.1038/ng.568 PMC2997674

[32] Dreja T, Jovanovic Z, Rasche A, Kluge R, Herwig R, et al (2010) Diet-induced gene expression of isolated pancreatic islets from a polygenic mouse model of the metabolic syndrome. Diabetologia 53: 309–320. 19902174 10.1007/s00125-009-1576-4 PMC2797618

[33] Novembre J, Di Rienzo A (2009) Spatial patterns of variation due to natural selection in humans. Nat Rev Genet 10: 745–755. 19823195 10.1038/nrg2632 PMC3989104

[34] Diamond J (2003) The double puzzle of diabetes. Nature 423: 599–602. 12789325 10.1038/423599a

[35] Fajans SS, Bell GI, Polonsky KS (2001) Molecular mechanisms and clinical pathophysiology of maturity-onset diabetes of the young. N Engl J Med 345: 971–980. 11575290 10.1056/NEJMra002168

[36] Chan JC, So W, Ma RC, Tong PC, Wong R, et al (2011) The Complexity of Vascular and Non-Vascular Complications of Diabetes: The Hong Kong Diabetes Registry. Curr Cardiovasc Risk Rep 5: 230–239. 21654912 10.1007/s12170-011-0172-6 PMC3085116

[37] Liu KH, Chan YL, Chan WB, Chan JC, Chu CW (2006) Mesenteric fat thickness is an independent determinant of metabolic syndrome and identifies subjects with increased carotid intima-media thickness. Diabetes Care 29: 379–384. 16443891 10.2337/diacare.29.02.06.dc05-1578

[38] Ozaki R, Qiao Q, Wong GW, Chan MH, So WY, et al (2007) Overweight, family history of diabetes and attending schools of lower academic grading are independent predictors for metabolic syndrome in Hong Kong Chinese adolescents. Arch Dis Child 92: 224–228. 17088339 10.1136/adc.2006.100453 PMC2083404

[39] Hu C, Zhang R, Wang C, Ma X, Fang Q, et al (2009) A genetic variant of G6PC2 is associated with type 2 diabetes and fasting plasma glucose level in the Chinese population. Diabetologia 52: 451–456. 19082990 10.1007/s00125-008-1241-3

[40] Park KS, Chan JC, Chuang LM, Suzuki S, Araki E, et al (2008) A mitochondrial DNA variant at position 16189 is associated with type 2 diabetes mellitus in Asians. Diabetologia 51: 602–608. 18251004 10.1007/s00125-008-0933-z

[41] Tan JT, Ng DP, Nurbaya S, Ye S, Lim XL, et al (2010) Polymorphisms identified through genome-wide association studies and their associations with type 2 diabetes in Chinese, Malays, and Asian-Indians in Singapore. J Clin Endocrinol Metab 95: 390–397. 19892838 10.1210/jc.2009-0688

[42] Ng MC, Park KS, Oh B, Tam CH, Cho YM, et al (2008) Implication of genetic variants near TCF7L2, SLC30A8, HHEX, CDKAL1, CDKN2A/B, IGF2BP2, and FTO in type 2 diabetes and obesity in 6,719 Asians. Diabetes 57: 2226–2233. 18469204 10.2337/db07-1583 PMC2494677

[43] Guan J, Zhao HL, Baum L, Sui Y, He L, et al (2009) Apolipoprotein E polymorphism and expression in type 2 diabetic patients with nephropathy: clinicopathological correlation. Nephrol Dial Transplant 24: 1889–1895. 19218599 10.1093/ndt/gfn734

[44] Zhao HL, Tong PC, Lai FM, Tomlinson B, Chan JC (2004) Association of glomerulopathy with the 5'-end polymorphism of the aldose reductase gene and renal insufficiency in type 2 diabetic patients. Diabetes 53: 2984–2991. 15504980 10.2337/diabetes.53.11.2984

[45] Purcell S, Cherny SS, Sham PC (2003) Genetic Power Calculator: design of linkage and association genetic mapping studies of complex traits. Bioinformatics 19: 149–150. 12499305 10.1093/bioinformatics/19.1.149

[46] Devlin B, Roeder K (1999) Genomic control for association studies. Biometrics 55: 997–1004. 11315092 10.1111/j.0006-341x.1999.00997.x

[47] Bax L, Yu LM, Ikeda N, Tsuruta H, Moons KG (2006) Development and validation of MIX: comprehensive free software for meta-analysis of causal research data. BMC Med Res Methodol 6: 50. 17038197 10.1186/1471-2288-6-50 PMC1626481

