# Supplementary material for: Familial Young-Onset Diabetes, Pre-Diabetes and Cardiovascular Disease Are Associated with Genetic Variants of DACH1 in Chinese
Source: PLoS One. 2014 Jan 20;9(1):e84770. doi: 10.1371/journal.pone.0084770 (PMC3896349; doi:10.1371/journal.pone.0084770)
Supplement: Table S1 — Summary of bioinformatics analysis of rs1408888 of DACH1 (Ch13:70910099-71339331) based on NCBI Build 36. (DOC) [file pone.0084770.s001.doc]

**Table S1.** Summary of bioinformatics analysis of rs1408888 of *DACH1* (Ch13:70910099-71339331) based on NCBI Build 36**.**

| **Feature** | **Position**  **(**NCBI Build 36**)** | **Result** | **Notes** |
| --- | --- | --- | --- |
| rs1408888 | Chr13:  71326648 | T/G polymorphism |  |
| GC% (±250bp) | Chr13: 71326148-71327148 | 33.8% |  |
| CpG island (±250bp) | Chr13: 71326148-71327148 | No |  |
| Transcription factor (TF) binding sites (±250bp) | Chr13: 71326148-71327148 | With 5% dissimilarity allowed, 31 TF binding sites were identified in the rs1408888 ±250bp region; TFIID and FOXP3 binding sites are common for both alleles. HNF-1A binding site is unique for T-allele; TBP, GR and C/EBPβ binding sites are unique for the G-allele. | Analysis by PROMO 3.0 using TRANSFAC  v.8.3 |
| FAIRE peaks | Chr13: 71326332-71326759 | Peak point 71326503; Score 637; SignalValue 0.017; P-value 0.063  145bp away from rs1408888 with a peak of pancreatic islets FAIRE signal | Giresi and Lieb Methods. (2009) 48:233-9. |
| DNaseI hypersensitive site |  | No | Stitzel, M L. et al. Cell Metab. (2010) 12:443-55. |
| Open chromatin (H3K4me3 association) | Chr13:  71335703-71340218 | 9Kb downstream from rs1408888 | Stitzel, M.L. et al. Cell Metab. (2010) 12:443-55. |
| CTCF binding sites | Chr13:  71351271-71351803 | About 25Kb downstream from rs1408888 | Stitzel, M.L. et al. Cell Metab. (2010) 12:443-55. |
| Islet-selective Clusters of Open Regulatory Elements (COREs) | Chr13:  70910039-71343328 | Lies on the 433Kb islet FAIRE CORE | Gaulton, K.J. et al. Nature Genet. (2010) 42:255-61. |
| Transcriptional module predicted by PReMod | Chr13:  71326382-71327308 | 4 modules predicted by PReMod in the vicinity of rs1408888 and one of them (mod030758) overlaps with rs1408888 region | Blanchette, M. et al. Genome Res. (2006) 16:656-68. |

| Conserved non-coding elements (CNE) | Chr13:  71324946-71325262 (hg18) | 1.5Kb upstream from rs1408888 with highly conserved non-coding element (CNE803) | Woolfe, A. et al. PLoS Biology (2005) 3:e7. |
| --- | --- | --- | --- |
| Regulatory elements | Chr13:  71323788-71326336 | OREG0002711 is 312 bp from rs1408888 | Open regulatory annotation database |
| Copy number variation (CNV) | Chr13:  71226326-71383406 | rs1408888 lies on the 15Kb CNV Variation_3912 | Redon R. et al. Nature (2006) 444:444-54. |
| microRNA | Chr13:  65690383-65690457 | hsa-mir-4704 is the closest microRNA 5.6Mb from rs1408888 |  |

**Abbreviations:** CTCF, CCCTC-binding factor, a DNA binding factor involved in gene insulation activity; FAIRE, Formaldehyde assisted isolation of regulatory elements, a technique which isolates DNA elements with open chromatins; FAIRE-CORE, Cluster of open regulatory elements isolated by the FAIRE technique; TFIID, transcription factor IID, a core component for transcription initiation; FOXP3, a forkhead box protein P3, a zinc finger transcription factor; HNF-1A, hepatic nuclear factor 1α, a homeo domain transcription factor; GR, glucocorticoid receptor, a zinc finger transcription factor; TBP, TATA-binding protein, binds to the TATA-box at the promoter; C/EBPβ, CCAAT/enhancer-binding protein β, a transcription factor binds to the CCAAT sequence to enhance gene expression.
